# Exacerbation of Brain Injury by Post-Stroke Exercise Is Contingent Upon Exercise Initiation Timing

**DOI:** 10.3389/fncel.2017.00311

**Published:** 2017-10-05

**Authors:** Fengwu Li, Xiaokun Geng, Hajra Khan, John T. Pendy Jr., Changya Peng, Xiaorong Li, Jose A. Rafols, Yuchuan Ding

**Affiliations:** ^1^Department of Neurology, Luhe Hospital, Capital Medical University, Beijing, China; ^2^Department of Neurosurgery, Wayne State University School of Medicine, Detroit, MI, United States; ^3^Department of Anatomy and Cell Biology, Wayne State University School of Medicine, Detroit, MI, United States

**Keywords:** infarction, apoptosis, brain metabolism, ATP, NAD, reactive oxygen species

## Abstract

Accumulating evidence has demonstrated that post-stroke physical rehabilitation may reduce morbidity. The effectiveness of post-stroke exercise, however, appears to be contingent upon exercise initiation. This study assessed the hypothesis that very early exercise exacerbates brain injury, induces reactive oxygen species (ROS) generation, and promotes energy failure. A total of 230 adult male Sprague-Dawley rats were subjected to middle cerebral artery (MCA) occlusion for 2 h, and randomized into eight groups, including two sham injury control groups, three non-exercise and three exercise groups. Exercise was initiated after 6 h, 24 h and 3 days of reperfusion. Twenty-four hours after completion of exercise (and at corresponding time points in non-exercise controls), infarct volumes and apoptotic cell death were examined. Early brain oxidative metabolism was quantified by examining ROS, ATP and NADH levels 0.5 h after completion of exercise. Furthermore, protein expressions of angiogenic growth factors were measured in order to determine whether post-stroke angiogenesis played a role in rehabilitation. As expected, ischemic stroke resulted in brain infarction, apoptotic cell death and ROS generation, and diminished NADH and ATP production. Infarct volumes and apoptotic cell death were enhanced (*p* < 0.05) by exercise that was initiated after 6 h of reperfusion, but decreased by late exercise (24 h, 3 days). This exacerbated brain injury at 6 h was associated with increased ROS levels (*p* < 0.05), and decreased (*p* < 0.05) NADH and ATP levels. In conclusion, very early exercise aggravated brain damage, and early exercise-induced energy failure with ROS generation may underlie the exacerbation of brain injury. These results shed light on the manner in which exercise initiation timing may affect post-stroke rehabilitation.

## Introduction

While advances in medical technologies over the last decade have significantly increased the survival rate of stroke patients, stroke remains a leading cause of major disability (Geng et al., 2012; Koronowski and Perez-Pinzon, 2015; Jiang and Duong, 2016; Mozaffarian et al., 2016). Among strategies to ameliorate physical disability after stroke, the use of post-injury, exercise-mediated adaptations is an emerging arena in neuroprotective rehabilitation (Arya et al., 2011). Evidence from both human and animal studies has suggested that physical activity and exercise enhance neuroplasticity, which ultimately improves functional outcomes. However, no optimal rehabilitation method for enhancing recovery is currently available in clinical settings, and present rehabilitation procedures are limited in their ability to reverse physical de-conditioning and optimize motor function (Ivey et al., 2006). Fundamental questions regarding initiation time, intensity and type of exercise, factors all known to affect rehabilitation, remain unanswered.

Among these questions, the influence of early (within hours) rehabilitation on functional outcomes after stroke has received substantial attention. Although current guidelines in many clinical practices recommend early mobilization after stroke, studies examining the effects of early exercise have yielded conflicting results. For example, Phase I of “A Very Early Rehabilitation Trial” (AVERT) suggested that mobilization of patients as early as 24 h after stroke is not only safe and associated with improved odds of favorable outcomes, but also cost effective (Bernhardt et al., 2006). However, neither the beneficial effects of early mobilization nor rationale explaining the underpinnings of such outcomes were presented in Phase II of AVERT. The completion of AVERT’s large-scale clinical study in 2014 painted a different picture than their preliminary results (Bernhardt et al., 2015). The study concluded that very early mobilization significantly reduced the odds of favorable outcome 3 months after stroke as compared to patients subjected to low dose, usual care settings. Additionally, the trial discovered no significant differences in immobility-related complications between patients subjected to very early mobilization and those treated with usual care.

In experimental animal models of stroke, some studies have suggested a beneficial effect to exercise if initiated as early as 24 h after ischemic or hemorrhagic stroke (Park et al., 2010; Matsuda et al., 2011; Zhang et al., 2013). Furthermore, if initiated early, mild to moderate exercise (though not heavy) is thought to promote recovery from ischemic stroke in rats (Lee et al., 2009). In contrast, a vulnerable period during the early phase (within 24 h) post-stroke has also been recognized (Humm et al., 1998; Risedal et al., 1999), as training initiated 24 h after permanent focal brain ischemia was found to exacerbate cortical tissue loss (Risedal et al., 1999). In addition, increased injury was detected in the forelimb area of the sensorimotor cortex of ischemic rats forced to overuse the impaired forelimb for 7 or 15 days post-injury (Kozlowski et al., 1996; Humm et al., 1998). Together, these studies have highlighted the importance of post-stroke exercise timing in recovery from injury and physical disability.

In the present study, we directly examined the effect of post-stroke exercise initiation timing on brain injury. In rats subjected to 2-h middle cerebral artery (MCA) occlusion, we evaluated the extent of brain injury by examining infarct volume and apoptotic cell death. The effects of two different exercise intensities on brain damage after stroke were compared. Furthermore, we assessed metabolic energy failure, which may play a key role in cellular injury, by examining reactive oxygen species (ROS) levels, ATP production and NADH levels. Protein expressions of angiogenic growth factors were also measured in order to determine whether post-stroke angiogenesis played a role in rehabilitation. Ultimately, we found that very early exercise enhanced brain injury, and early exercise-induced energy failure with ROS generation may underlie exacerbation of brain injury.

## Experimental Procedures

A total of 230 adult male Sprague-Dawley rats (280–300 g, Vital River Laboratory Animal Technology Co., Ltd., Beijing, China) were used in this study. The protocol was approved by the Animal Care and Use Committee, Capital Medical University, Beijing, China, and was in accordance with the National Institutes of Health (USA) Guide for the Care and Use of Laboratory Animals. Careful measures were taken to minimize animal suffering and the number of animals used. Animals were randomly divided into eight groups, including two sham injury control groups (*n* = 10 per group), nine stroke groups not subjected to post-stroke exercise (*n* = 10 per group) and 12 stroke groups subjected to post-stroke exercise initiated after either 6 h (*n* = 40), 24 h (*n* = 40), or 3 days (*n* = 40) of reperfusion or equivalent time. All animals were sacrificed at the same time points: 24 h after forced exercise for detection of infarct volume, 30 min after exercise for quick detection of levels of ATP, NADH and ROS, 24 h for cell death detection, as well as at 3 h and 24 h after exercise termination for later protein expression of angiogenic growth factors. All of the time points for animal sacrifice from this study were based on previous studies (Kochanski et al., 2013; Geng et al., 2016; Shen et al., 2016; Li et al., 2017). The mortality rate was low (less than 10%) and was about equal between paired groups (i.e., stroke groups with or without exercise). The death of ischemic rats in the present study was caused by the operative skills and skull base hemorrhage due to arterial rupture during filament insertion, rather than the ischemic time. All animals were randomly divided into different groups, and all data sets were analyzed in a blind manner.

### Focal Cerebral Ischemia

The induction of focal cerebral ischemia has been described previously (Dang et al., 2011; Wang et al., 2012). Briefly, rats were initially anesthetized with 1%–3% isoflurane and a mixture of 70% nitrous oxide and 30% oxygen, and were maintained with a facemask using 1% isoflurane delivered from a calibrated precision vaporizer. Poly-L-lysine-coated intraluminal nylon (4.0) sutures were used to yield consistent infarcts, greatly reducing inter-animal variability. During the unilateral, 2-h MCA occlusion procedure, blood pCO_2_ and pO_2_, mean arterial pressure and rectal temperature were monitored continuously. Rectal temperature was maintained between 36.5°C and 37.5°C using a circulating heating pad and a heating lamp. For subsequent examination of all markers (cell death, ROS, ATP, NADH, protein expression), the MCA-supplied regions in affected cerebral hemispheres, including the ischemic core and penumbra, were analyzed. After MCA occlusion, the modified scoring systems (5 scores) initially proposed by Longa et al. (1989) for neurological deficits were used to confirm brain injury. If the scores were 1 or below, the MCA occlusions were considered unsuccessful and the rats were excluded from further studies. In our study, about 10% of animals with MCAO were discarded for this reason.

### Rotarod Exercise

The rotarod (R03-1; Xin-Ruan Instruments, Inc., Shanghai, China) was used as a training platform in this study. The rod is 7 cm in diameter, 11 cm in length and covered with smooth rubber. Animals performed 30 min of rotarod exercise. In intense exercise, speed incrementally increased from 5 rpm to 35 rpm over the 30 min period, while in mild exercise, speed incrementally increased from 5 rpm to 15 rpm over the 30 min period. Exercise was initiated after 6 h, 24 h, or 3 days of reperfusion. To identify the effect of exercise intensity on brain damage after stroke, infarct volumes were measured 24 h after both mild and intense exercise completion. For all other experiments, intense exercise settings were used. The apparatus delivered an electric shock (0.1 mA, 3 s) to animals that fell from the rotating cylinder, forcing the animals to exercise continuously for the duration of the exercise period. During the study, animals from different exercise groups only occasionally fell from the rod and did not suffer from any additional injuries. We found that the spontaneous recovery of rats at 6 h to be well enough for such an exercise protocol, and comparable to recovery on the rotarod at later time points. For pre-conditioning to rotarod exercise, rats were required to perform rotarod training at a constant speed (15 rpm) for 20 min/day for 3 days prior to MCA occlusion. Both non-exercise and exercised ischemic animals were housed in groups of three in standard cages for an equal amount of time. In our study, rats ate and drank normally 1 h after MCA occlusion. Although typical motor function impairment (movement in a circle) was observed, the animals moved around in the cage and were able to run on the rotarod apparatus for 30 min.

Although several drops were found during that period, the electric shock would keep rats running. The electric shock provided to rats that fell from the rotarod may have induced a stress response, potentially confounding our results and overstating the effect of early exercise. The influence of electric shock on cerebral infarct volumes has been addressed by our previous work (Hayes et al., 2008), indicating that forced exercise (exercise + shock) groups exhibited smaller infarct volumes than voluntary exercise groups and groups that were only exposed to electric shock. Furthermore, a newly published systematic review concluded that forced exercise consistently reduces lesion volumes and is protective against oxidative damage and inflammation (Austin et al., 2014). Together, these results suggest that the electric shock employed in this study likely had a minimal role in exacerbating the effect of very early exercise on post-ischemic brain injury.

### Cerebral Infarct Volume

After 30 h of reperfusion or 24 h of exercise, brains were resected from ischemic rats and cut into 2 mm-thick slices (brain matrix) and treated with 2,3,5-triphenyltetrazolium chloride (TTC; Sigma-Aldrich, St. Louis, MO, USA) for staining. An indirect method for calculating infarct volume was used to minimize error caused by edema (Wang et al., 2012).

### Apoptotic Cell Death Detection by ELISA

Previous research on voluntary exercise in rats has suggested that exercise-induced central nervous system apoptosis peaks after 24 h (Kerr and Swain, 2011). We therefore chose to examine post-exercise apoptosis 24 h after completion of exercise regimens, and at equivalent time points in non-exercise groups. For quantification of apoptosis-related DNA fragmentation at 24 h after exercise, a commercial enzyme immunoassay (Cell Death Detection ELISA; Roche Diagnostics, Indianapolis, IN, USA) was used to identify and quantify cytoplasmic histone-associated DNA fragments, as described previously by us (Fu et al., 2013).

### TUNEL Assay

Animals were sacrificed with cardiac perfusion of saline followed by 4% paraformaldehyde in 0.1 M phosphate buffer (PB), pH 7.4. Coronal brain frozen sections were cut on a microtome at a thickness of 30 mm from +2.0 mm to −4.0 mm of Bregma. The DNA fragmentation was determined by TUNEL assay by using a commercially available kit (*In situ* Cell Death Detection Kit, Fluoresce, Roche, Indianapolis, IN, USA). Briefly, the fixed slides were washed three times for 5 min with PBS and permeabilized with 0.1% (v/v) Triton X-100 containing 0.1% (w/v) sodium citrate for 2 min on ice. Samples were then incubated in 50 μl of TUNEL reaction mixture for 1 h at 37°C. Slides were washed three times with PBS. Positive TUNEL staining was observed under a fluorescence microscope (DM4000, Leica, Germany). The TUNEL index was determined by counting the positive stained cells in all cells.

### Angiogenic Protein Expression

Protein expression of angiopoietin-1 (Ang-1), angiopoietin-2 (Ang-2) and vascular endothelial growth factor (VEGF) was measured at 3 and 24 h after exercise termination or equivalent time points in the non-exercise group using Western blot as previous described (Shen et al., 2016). The angiopoietin family (Ang-1 and Ang-2) plays a key role in neurovascular remodeling as it is involved in maturation, stabilization and remodeling of vessels (Ding Y.-H. et al., 2004). VEGF is a known inducer of angiogenesis through the formation of immature vessels and has been implicated as a neuroprotective agent in both hypoxia and glucose starvation-induced compensatory neovascularization (Ding Y.-H. et al., 2004; Ding et al., 2006). Upon conclusion of electrophoresis, proteins were transferred to a polyvinylidene fluoride membrane. Membranes were incubated with a primary antibody (rabbit polyclonal anti-Ang-1, 1:500, R&D Systems, Minneapolis, MN, USA; rabbit polyclonal anti-Ang-2 antibody, 1:500, R&D Systems, Minneapolis, MN, USA; rabbit polyclonal anti-VEGF, 1:1000, Santa Cruz, Dallas, TX, USA) for 24 h at 4°C. Next, membranes were washed three times with PBS for 6 min each, and re-incubated with a secondary antibody (goat anti-rabbit IgG, Santa Cruz Cat) for 1 h at room temperature. An ECL-system was used to detect immunoreactive bands by luminescence. Western blot images for each antibody, including β-actin, were analyzed using an image analysis program (ImageJ 1.42, National Institutes of Health, USA) to quantify protein expression in terms of relative image density. The mean amount of protein expression from the control group after stroke was assigned a value of 1 to serve as reference.

### ROS Production

In order to identify the immediate effect of exercise on metabolism, we examined ROS production 0.5 h after completion of exercise interventions in exercise groups, and at equivalent time points in non-exercise groups. The ROS method, as described previously by us (Geng et al., 2013), tests for H_2_O_2_ with hydrogen peroxidase linked to a fluorescent compound. This is a reliable method for superoxide determination, as it assesses the enzymatic dismutation of superoxide into H_2_O_2_. Briefly, brain tissue was homogenized in buffer (mannitol 225 mM, sucrose 75 mM, EGTA 1 mM, HEPES 5 mM at pH 7.4, 1 mg/ml fatty acid free BSA, and 0.1% Triton-X-100). We employed the bicinchoninic acid assay for determination of protein concentration. Samples were diluted to 10 mg/ml, to which 100 μg/ml digitonin was added. After a 30 min incubation, H_2_O_2_ in brain homogenates was detected using 50 μM Amplex Red (Amplex Red Reagent; Thermo Fisher Scientific, Waltham, MA, USA), 0.1 U/ml horseradish peroxidase, and respiratory substrates (4 mM pyruvate, 2 mM malate, 2 mM glutamate and 0.8 mM complex V inhibitor oligomycin) at 37°C. Fluorescence (*λ*_ex_ = 535 nm, *λ*_em_ = 595 nm) was recorded every 30 s for 30 min on a multimode detector (DTX-880; Beckman Coulter, Inc., Fullerton, CA, USA).

### ATP Assay

We employed an ATP assay kit (ATP Colorimetric/Fluorometric Assay Kit; Bio Vision, Inc., Mountain View, CA, USA) to examine brain ATP levels. We measured ATP levels 0.5 h after completion of exercise interventions in exercise groups, and at equivalent time points in non-exercise groups. Briefly, brain tissue samples (12 mg) were homogenized with an ATP Assay Buffer, and centrifuged at 12,000 *g* for 10 min. The supernatant and gradient ATP standards were individually mixed with ATP Reaction Mix. After a 30 min incubation avoiding light, ATP levels were quantified (optical density = 570 nm).

### NADH Assay

We employed an NADH assay kit (NAD/NADH Quantitation Colorimetric Kit; Bio Vision, Inc., Mountain View, CA, USA) to examine brain NADH levels according to the manufacturer’s protocol. We measured NADH levels 0.5 h after completion of exercise interventions in exercise groups, and at equivalent time points in non-exercise groups. Briefly, brain tissue samples (20 mg) were homogenized with NADH/NAD Extraction Buffer, and subsequently centrifuged at 14,000 *g* for 5 min. One fraction underwent heat treatment at 60°C for 30 min and was subsequently used to assay NAD^+^ content. The other fraction was used to assay total NAD (NADH and NAD^+^) without heat treatment (optical density = 450 nm).

### Statistical Analysis

**Figure 1:**
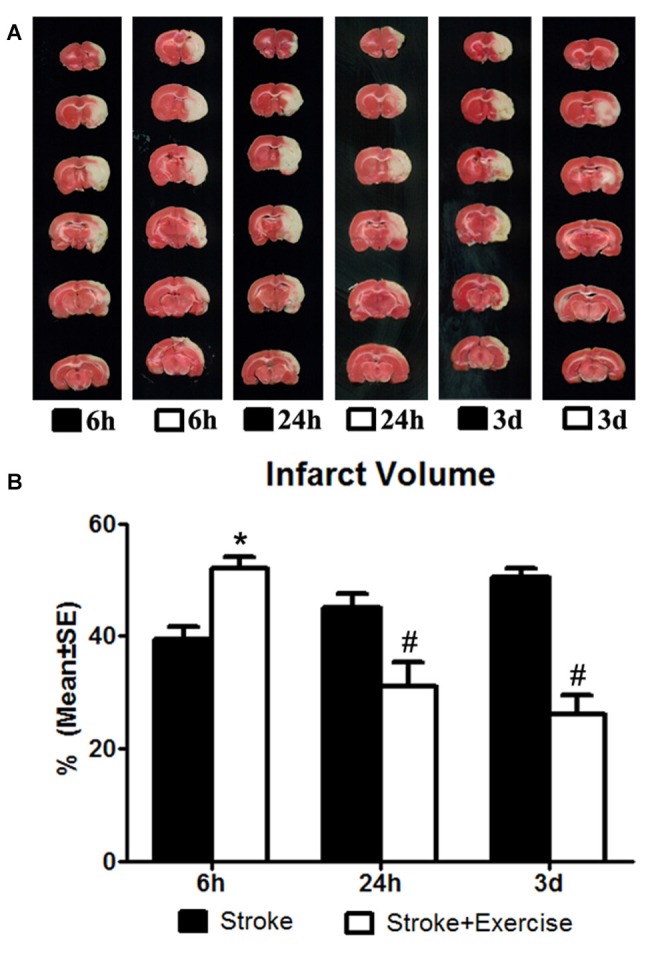
**(A)** 2,3,5-Triphenyltetrazolium chloride (TTC) staining illustrating infarct volumes in ischemic rats (subjected to 2-h middle cerebral artery (MCA) occlusion) with or without exercise initiated at 6 h, 24 h and 3 days after reperfusion. **(B)** In contrast to the non-exercise groups, very early exercise at 6 h largely (*p < 0.05) increased neural damage, while the later exercise groups conducted at 24 h and 3 days after reperfusion demonstrated a significant (^#^*p* < 0.05) reduction in apoptosis.

**Figure 2:**
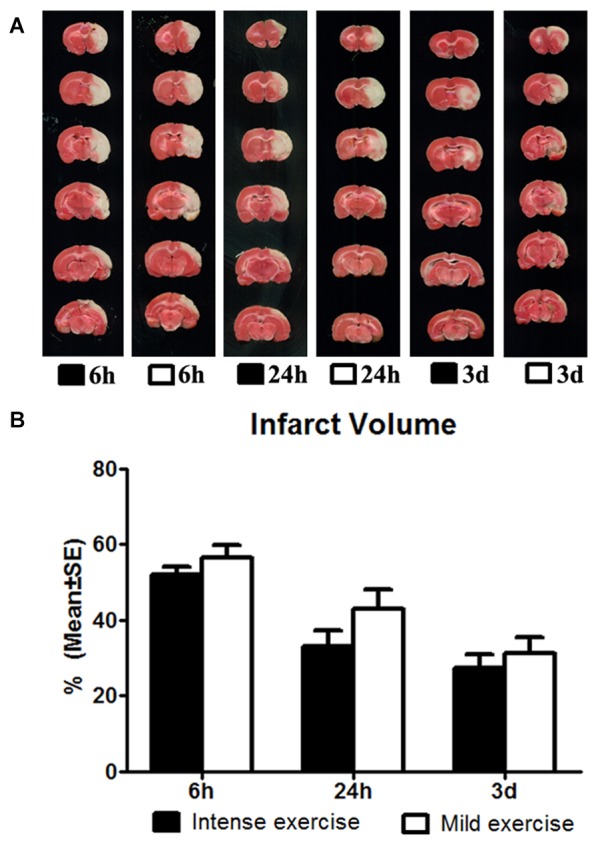
**(A)** Comparison of mild and intense exercise (initiated at 6 h, 24 h and 3 days of reperfusion) on infarct volume determined by TTC. **(B)** Compared to the intense exercise groups, infarct volumes of the mild exercise groups were slightly but not significantly increased after all exercise time points.

**Figure 3:**
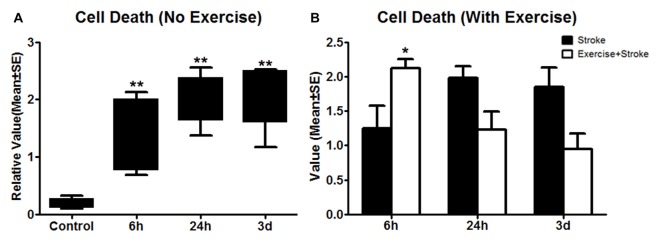
**(A)** Apoptotic cell death detected using ELISA was measured 24 h after exercise termination or the equivalent time point in non-exercise animals. One-way ANOVA of non-exercise groups indicated a significant (**p < 0.01) increase in all non-exercise ischemic rat groups (6 h, 24 h and 3 day) compared to sham-operation controls. **(B)** In contrast to non-exercise groups, The student *t*-test indicates that very early exercise initiated at 6 h of reperfusion significantly (*p < 0.05) increased cell death, while groups in which exercise was initiated after 24 h and 3 days of reperfusion exhibited non-significant reductions in apoptosis.

**Figure 4:**
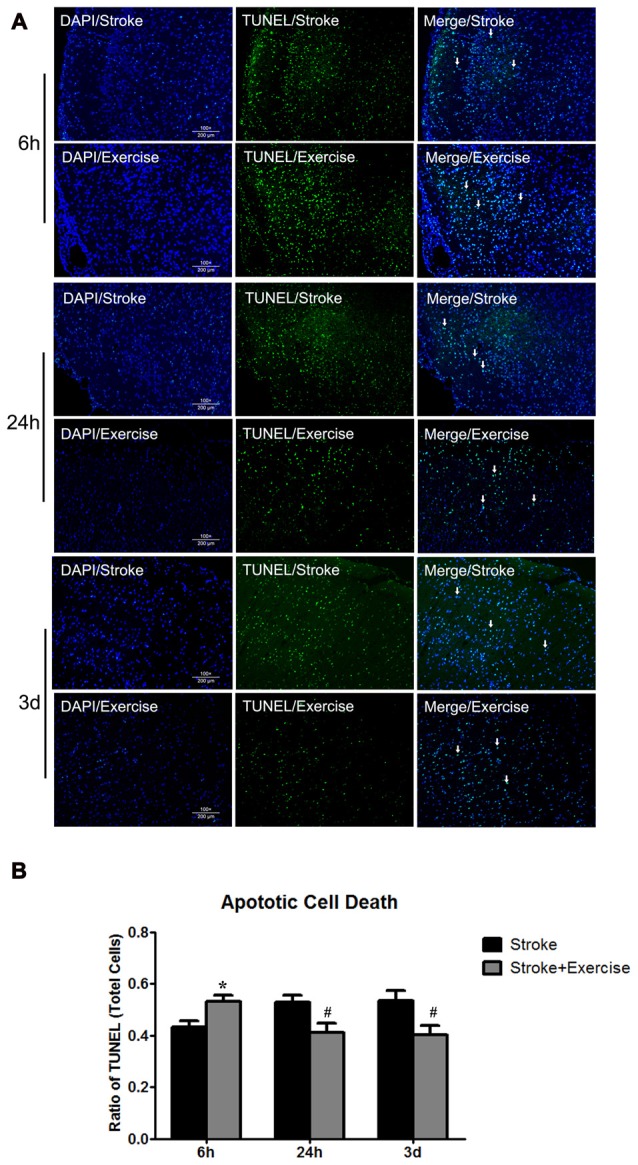
**(A)** TUNEL of non-exercise and exercise groups (initiated at 6 h, 24 h and 3 days of reperfusion). **(B)** Compared to stroke groups without exercise, apoptotic cell death was significantly (*p < 0.05) increased after very early exercise (initiated at 6 h of reperfusion), while later exercise (24 h and 3 days) significantly (^#^*p* < 0.05) reduced cell death as compared to non-exercise stroke group.

**Figure 5:**
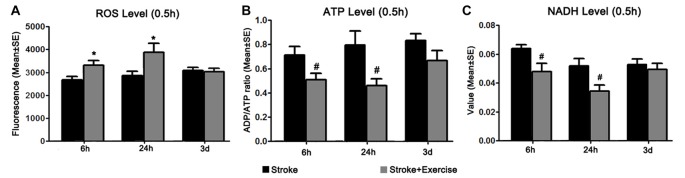
**(A)** Reactive oxygen species (ROS) levels were examined 0.5 h after exercise termination in ischemic rats with exercise and at equivalent time points in ischemic rats without exercise. Compared to sham controls (reference as 1, not shown), ischemic animals exhibited significantly increased ROS levels. Further increases in ROS production were observed in the early (6 h and 24 h, *p < 0.05) exercise groups as compared to non-exercise controls. Late exercise (initiated after 3 days of reperfusion) did not have a significant impact on ROS levels, as compared to non-exercise ischemic rats. **(B)** ATP levels were examined 0.5 h after exercise termination in exercise groups and at equivalent time points in non-exercise controls. MCA occlusion significantly decreased ATP production as compared to sham controls (reference as 1, not shown). Early exercise groups (6 h and 24 h, ^#^*p* < 0.05), though not the late exercise group (3 days), exhibited further reductions in ATP levels as compared to non-exercise controls. **(C)** NADH levels, measured 0.5 h after exercise termination in exercise groups and at equivalent time points in ischemic rats, were significantly decreased in all non-exercise ischemic groups as compared to sham control (reference as 1, not shown). Exercise initiated after 6 h and 24 h of reperfusion further reduced (^#^*p* < 0.05) NADH levels as compared to corresponding non-exercise groups.

**Figure 6:**
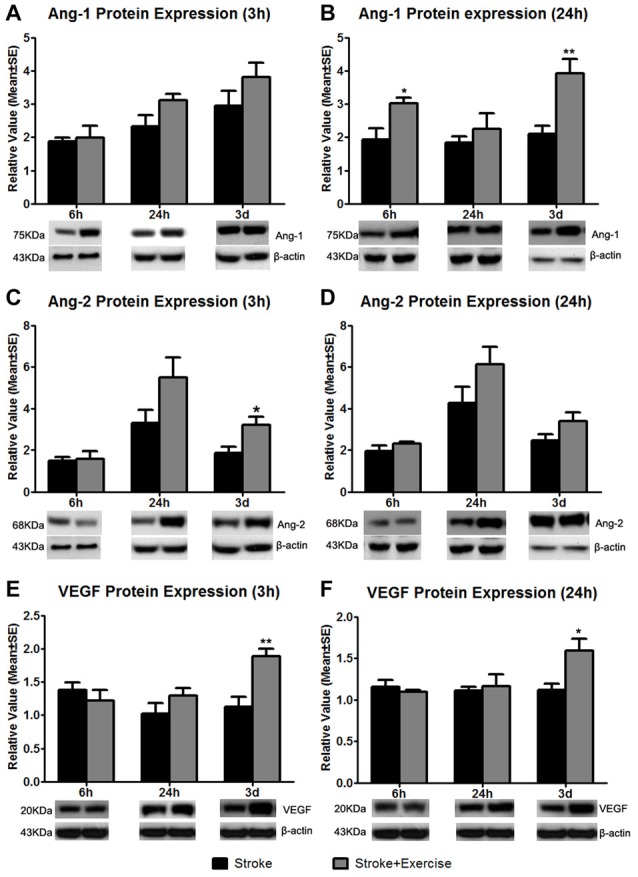
Based on sham control (reference as 1, not shown), angiopoietin-1 (Ang-1) expression at 3 h after exercise termination was slightly increased in exercise groups compared to non-exercise ischemic rat groups at 6 h, 24 h and 3 days after reperfusion **(A)**. A significantly enhanced increase in Ang-1 protein expression at 24 h after exercise termination was observed in the very early (6 h; *p < 0.05) and late (3 days; **p < 0.01) exercise groups while a slight increase was seen in the 24 h exercise group **(B)**. Angiopoietin-2 (Ang-2) protein expression was measured 3 and 24 h after exercise termination or the equivalent period **(C,D)**. At 3 h, late exercise (3 days) resulted in a significant (*p < 0.05) increase in Ang-2 protein expression compared to non-exercise ischemic rat groups, while no changes were seen with early exercise (6 and 24 h). For 24 h after exercise termination, exercise at three time points resulted in an increase in Ang-2 protein expression, although they did not reach a significant level. **(E)** Vascular endothelial growth factor (VEGF) protein expression was measured 3 h after exercise termination or the equivalent period. Only late exercise (3 days) significantly (**p < 0.01) increased VEGF protein expression compared to non-exercise ischemic rat groups. **(F)** Similar results were seen in which late (3 days) resulted in a significant (*p < 0.05) increase in VEGF protein expression. Representative immunoblots are presented.

Statistical analysis was performed with SPSS for Windows, Version 17.0 (SPSS, Inc., Chicago, IL, USA). Sample size in this study was determined based on a power analysis on preliminary and previous data, which suggested a sample size of 10 animals for each group for the present studies, in order to have power exceeding 95% (*α* = 0.05, power = 0.95) to yield statistically significant results (*p* < 0.05). All data were described as mean ± SE. Cell death among multiple groups without exercise were assessed by using one-way ANOVA and Duncan’s multiple range tests with significance levels at *p* < 0.05 (Figure 1A). Student *t*-test, with *p* < 0.05 (*), was used for all other comparisons in order to show the difference of outcome with or without exercise for each initiation time. Although results between exercise and non-exercise groups at different initiation time points (3 h, 24 h and 3 days) were demonstrated in the same figures (Figures 1B, 2B, 3B, 4B, 5, 6), no effort was given to statistically show their association.

## Results

### Physiological Parameters

Average values of blood pO_2_, pCO_2_ and pH in all ischemic rats upon termination of MCA occlusion were within normal ranges (Table 1). Body temperature was maintained at approximately 37°C throughout the course of surgery. No obvious differences in rates at which rats fell from the rota-rod apparatus were found between rats in which exercise was initiated after 6 h, 24 h, or 3 days of reperfusion.

**Table 1:** Physiological variables monitored during surgery.

	Prior to MCA occlusion	Onset of reperfusion	After reperfusion
pCO_2_ (*mm Hg*)	44.5 ± 1.2	43.3 ± 3.7	41.1 ± 5.7
pO_2_ (*mm Hg*)	134.6 ± 5.5	131.1 ± 6.6	129.2 ± 9.9
pH	7.40 ± 0.02	7.41 ± 0.03	7.39 ± 0.02

### Initiation and Intensity Effect of Exercise on Infarct Volume

The effect of exercise timing on brain damage after stroke was determined by infarct volumes (Figure 1). Compared to the stroke group without exercise, infarct volume determined by TTC was significantly (*p* < 0.05) increased after very early exercise (intensive, initiated at 6 h of reperfusion), while late exercise (24 h and 3 days) reduced (*p* < 0.05) infarct volume as compared. We further identified the effect of exercise intensity (intensive vs. mild) on brain damage after stroke, infarct volumes (Figure 2). As compared to the intense exercise group, mild exercise did not reduce any infarct volume but slightly (not significantly) increased the infarction in all exercise timings (initiated at 6 h, 24 h and 3 days of reperfusion).

### Apoptotic Cell Death

As compared to sham control, cell death, determined by ELISA, was elevated (*p* < 0.01) by ischemia/reperfusion injury in all the three non-exercise groups. This increased cell death was enhanced by very early exercise (6 h; *p* < 0.05), but not by the later ones (24 h, 3 day; Figure 3). In fact, cell death rates seemed to decrease as exercise was initiated further from reperfusion. In TUNEL assays (Figure 4), again as compared to the stroke group without exercise, apoptotic cell death was significantly (*p* < 0.05) increased after very early exercise (initiated at 6 h of reperfusion), while later exercise (24 h and 3 days) significantly reduced cell death as compared to non-exercise stroke groups. TUNEL as well as TTC and ELISA for apoptosis-related DNA fragmentation demonstrated similar results, indicating that very early exercise (6 h) increased neural damage.

### Brain Metabolism

In order to explain the underlying mechanism of early exercise on stroke-induced injury, we identified the effect of exercise initiation timing on brain oxidative metabolism, as quantified by ROS, NADH and ATP levels 0.5 h after completion of exercise interventions in the exercise groups, and at equivalent time points in non-exercise groups (Figure 5). ROS levels were significantly increased in all three non-exercise stroke groups (*p* < 0.01), as compared to sham control (reference as 1, not shown; Figure 5A). Additional increases in ROS levels were observed after early exercise (6 h, 24 h; *p* < 0.05) but not late exercise (3 days), suggesting that early exercise aggravates cellular injury by stimulating ROS generation. After stroke, ATP levels were significantly (*p* < 0.01) decreased in all three non-exercise ischemic groups after 6 h, 24 h and 3 days of reperfusion. This suggests a decrease in ATP production, an increase in ATP utilization, or both. Figure 5B shows that exercise at 6 h and 24 h, but not at 3 days, exacerbated (*p* < 0.05) the stroke-induced ATP decreases as compared to the non-exercise stroke group, indicating further energy failure. To further assess brain energy metabolism, we investigated the effect of exercise on cellular levels of NADH. Again, NADH levels were significantly reduced in all non-exercise ischemic rats (*p* < 0.01). Early exercise at 6 h and 24 h (*p* < 0.05), but not 3 day exercise further reduced NADH activity (Figure 5C).

### Expression of Angiogenic Factors

Compared to non-exercise groups, Ang-1 protein expression increased to variable degrees in all exercise groups (initiated 6 h, 24 h and 3 days after reperfusion) when measured 3 (Figure 6A) and 24 h (Figure 6B) after exercise completion. The increases in Ang-1 protein expression only reached a significant level with the very early exercise (6 h; *p* < 0.05) and the late exercise (3 days; *p* < 0.01) at 24 h after exercise. Similarly, compared to the non-exercise group, there were no changes in Ang-2 protein expression with very early exercise (6 h) group at either of the 3 (Figure 6C) and 24 h (Figure 6D) time periods after exercise termination. There was a slight increase in Ang-2 protein expression in the early exercise (24 h) group at 3 and 24 h after exercise. Late exercise (3 days) significantly (*p* < 0.05) increased the expression of Ang-2 at 3 h after exercise, while only slightly increasing Ang-2 expression 24 h after exercise. Compared to the non-exercise group, VEGF protein expression decreased slightly with the very early exercise (6 h) group when measured at both 3 (Figure 6E) and 24 h (Figure 6F) after exercise. There was a slight increase in VEGF protein expression in the early exercise (24 h) group at 3 and 24 h after exercise. Late exercise (3 days) significantly increased expression of VEGF measured at 3 h (*p* < 0.01) and 24 h (*p* < 0.05) after exercise. Taken together, there was a trend of increase in angiogenic factor expression, especially after late exercise and late termination.

## Discussion

The present study has demonstrated the time-sensitive effects of exercise on post-stroke brain injury by highlighting the metabolic alterations that are likely involved. We also identified the effects of exercise intensities on brain damage after stroke following intense and mild exercise. Our study demonstrated that at the early stage, exercise initiations but not intensities attenuated brain damage. Our study revealed that early (up to 24 h) post-stroke exercise increases apoptotic cell death and activity, enhances ROS production, and adversely affects energy status, while very early (6 h) post-stroke exercise clearly exacerbates brain infarction and cell death. The present data is in agreement with our earlier work (Ding et al., 2003; Li et al., 2004). Very early post-stroke exercise seems to exacerbate brain injury, while late exercise seems to be beneficial. Our present findings are consistent with the conclusions of a recent systematic review that recognized a short therapeutic exercise initiation time window, with exercise onset before 3 h or beyond 3 days providing little benefit in animal models (Austin et al., 2014). Although ATP measurements in lysates samples are not stable, by combining them with the results of NADH and ROS, ATP levels may be a meaningful addition in our study. In terms of cause-and-effect, early exercise may aggravate brain oxygen metabolism, thus impairing oxidative phosphorylation and subsequently leading to a decrease in ATP production. This study includes measurements of both penumbra and core ischemic regions. In our future study, we will separate the two regions for measurement in order to further determine how these parameters play a role in the fate of ischemic issue.

Accumulating evidence supports the notion that physical exercise after stroke may improve functional outcomes via the induction of neuronal plasticity. Previous laboratory studies using rat models have implemented exercise as early as 24–48 h post-ischemia (Lee et al., 2009; Matsuda et al., 2011), and have chosen 4 or 5 days post-stroke for initiation of relatively late exercise (Ding et al., 2003; Ding Y. et al., 2004; Tamakoshi et al., 2014). The earliest phase of exercise implementation in clinical trials (AVERT) is within 24 h (median time of 18.5 h) of stroke onset (Bernhardt et al., 2015). Comparable age data between rats and humans suggests that 24 h for an adult rat corresponds to ~31 days for an adult human (Sengupta, 2013). While this correlation may be imperfect, it raises doubt as to whether exercise initiation at 24 h in rats is early enough to simulate human conditions. Because of this particular variable, we opted to assess three post-reperfusion exercise initiation time points (6 h, 24 h and 3 days). While 6 h might be very early for clinical treatment, this time point may be more appropriate for mechanistic studies of post-stroke exercise in animal models. To our knowledge, this is the first study to use a spectrum of times to accurately determine optimal exercise initiation timing.

Mechanistically, the metabolic dysfunction caused by stroke may be compounded by that generated by early exercise. Our results suggest that exercise might be a double-edged sword, in which its beneficial effects on functional recovery might be blunted by a tendency to exacerbate tissue loss. Hypermetabolism, rather than hypometabolism, likely underlies the increased tissue loss (Kim and Yenari, 2015). When ischemia impairs the mitochondrial oxidative phosphorylation system, the brain compensates to meet metabolic needs through hyperglycolysis (Schurr, 2002), ultimately accelerating ROS generation through lactic acidosis and resulting in neuronal death (Chan, 2001). A recent study supports exercise-induced hypermetabolism in brain as demonstrated by increased mRNA and protein levels of glucose transporter 1 and 3 (GLUT1 and GLUT3), phosphofructokinase (PFK), lactate dehydrogenase (LDH) and phosphorylated adenosine monophosphate kinase (pAMPK; Kinni et al., 2011), all indicative of enhanced glucose metabolism. Elevated levels of GLUT1 and GLUT3, the key integral membrane glycoproteins facilitating glucose transport, increase the brain’s capacity to utilize glucose (Schubert, 2005). Lactate is considered a dead-end product of glycolysis under anaerobic conditions (Gladden, 2004). In hypoxic environments, LDH processes lactate and elevated levels of LDH are indicative of a hyperglycolytic state (Schurr, 2002; Rossignol et al., 2003). Additionally, exercise results in substantially increased levels of PFK-1, the key regulatory enzyme in glycolysis (Almeida et al., 2004). Elevated levels of PFK-1 are indicative of high-energy demand and consumption. Increased levels of both adenosine monophosphate kinase (AMPK) and pAMPK (the active form of AMPK) after exercise are suggestive of both enhanced catabolism (Schurr, 2002) and increased glucose uptake (Carling, 2004). In addition, exercise-induced increases in cerebral blood flow (CBF; Ide and Secher, 2000; Liebeskind, 2015) further indicate high metabolic demands related to activity. Consistently, animals that are placed in enriched environments (Black et al., 1991) or that engage in sustained motor activity (Black et al., 1990; Isaacs et al., 1992; Ding Y. et al., 2004; Ding Y.-H. et al., 2004; Ding et al., 2006) have significantly higher vascular densities than non-enriched, non-active rats. Importantly, clinical studies in stroke patients conducted in the early 1970s first reported that the energy requirements of patients with hemiparetic gait were elevated by 55%–100% compared to normal controls (Corcoran et al., 1970; Gersten and Orr, 1971). In animal studies, hypoxic stress and forced post-stroke physical exercise result in an increased demand for ATP-generating capacity and elevated glucose metabolism in the brain (Kinni et al., 2011; Dornbos et al., 2013). Together, these results indicate that exercise increases the metabolic activity of the brain, driving it to consume more energy via glycolysis.

Exercise after stroke has been shown to induce angiogenesis and improve functional outcomes in both animal models and human patients (Zheng et al., 2011; Ergul et al., 2012). Our data demonstrates that early exercise makes no difference in post-stroke angiogenesis. While it is clear that angiogenesis genes were up-regulated in post-stroke exercise groups compared to non-exercise ischemic rat groups (Ergul et al., 2012), no significant changes in protein expression were observed in any of the very early (6 h) exercise groups apart from Ang-1 protein expression at 24 h after exercise termination. The results do suggest that there may be a possibility of late exercise (3 days) inducing some degree of angiogenesis, as demonstrated by significant rise in VEGF levels at 3 h and 24 h after exercise termination, Ang-1 levels at 24 h and Ang-2 levels at 3 h after exercise termination. However, it is very improbable that the angiogenic growth factor response truly lead to the formation of blood vessels, as well as function of newly formed vessels, as this is unlikely to have occurred within 3 days. Overall, our findings indicate that angiogenic factors do not play a major role in an early protocol, while late exercise may exert a beneficial effect on protein expression.

In summary, this study demonstrates the time-sensitive effects of exercise on stroke-induced brain injury, and contributes to the understanding of the metabolic mechanisms underlying post-stroke tissue injury. The data support an exercise initiation time point of 1–3 days post-reperfusion in order to enhance the benefits of exercise, while concurrently avoiding the detriments that may hamper functional outcomes. The results of the present study may ultimately lead to the development of an effective exercise-based stroke rehabilitation regimen.

## Author Contributions

The authors sincerely thank Dr. Zongjian Liu for his assistance in conducting the experiments. FL, CP and XL conducted the animal and biochemical experiments employed in this research. FL, XG, HK, JTP and JAR were instrumental in preparing and revising the manuscript. YD was responsible for the experimental design, in addition to assisting with manuscript preparation and revision.

## Conflict of Interest Statement

The authors declare that the research was conducted in the absence of any commercial or financial relationships that could be construed as a potential conflict of interest.
